# Emotional sociology applied: predictive influence of affective neuroscience personality traits on Chinese preschool teachers’ performance and wellbeing

**DOI:** 10.3389/fpsyg.2024.1372694

**Published:** 2024-05-31

**Authors:** Ling Lu, Lu Jian

**Affiliations:** ^1^Faculty of Education, Southwest University, Chongqing, China; ^2^College of Teacher Education, Xichang University, Xichang, China; ^3^Faculty of Psychology, Southwest University, Chongqing, China

**Keywords:** affective neuroscience, chain mediation model, emotional tendencies, job performance, job satisfaction, preschool education, teacher engagement, work engagement

## Abstract

**Background:**

The interplay between teaching engagement and performance has garnered attention in both theoretical and empirical research, primarily due to its influence on student academic achievement, teacher well-being, and the realization of institutional goals. This is especially pertinent in the realm of preschool education, where the scope of learning extends beyond academic content to encompass the broader socialization of children. Drawing from Affective Neuroscience research, this study investigates the role of affective tendencies as mediators in the relationship between work engagement and job performance.

**Objective:**

The primary aim of this research is to examine a chain mediation model that hypothesizes the predictive role of teacher engagement. This model posits the intermediary influence of four basic emotions—CARING, SEEKING, ANGER, and FEAR—followed by the mediating effect of job satisfaction on teacher job performance.

**Method:**

The study utilized a sample of 842 Chinese preschool teachers. Data were collected through an online questionnaire, employing a time-lagged design. The analysis was conducted using Model 80 of the PROCESS Macros.

**Results:**

The findings reveal that both positive and negative emotions significantly predict teachers’ job satisfaction. However, job satisfaction does not influence job performance. The analysis confirmed the direct and total effects of teacher engagement, as well as the indirect effects, particularly through the positive emotion of Caring.

**Implications:**

The results are instrumental in informing and refining interventions designed to enhance teacher engagement and performance, underscoring the importance of emotional factors in the educational environment.

## Introduction

Work engagement among Chinese preschool teachers holds a pivotal role in fostering development on both an individual and societal scale (Ji and Cui, 2021). Despite recognition of its influence on emotional well-being, job satisfaction, teacher effectiveness, and retention rates, existing literature has not thoroughly delineated the intricate dynamics that underlie these effects (Juliana et al., 2021). While the impact of teacher engagement on various job-related outcomes has been explored, the literature remains scant on how specific emotional experiences and job satisfaction mediate this relationship.

Furthermore, while the Affective Neuroscience Theory, as developed by Panksepp between 1992 and 2006, provides a robust framework for understanding the primary emotional systems integral to psychological health and their implications for behavior, its application in educational settings, especially in the context of teacher work engagement, remains underexplored (Panksepp, 1982, 2004, 2005, 2013). The theory’s potential to illuminate the biological underpinnings of teacher emotions and their management within educational environments suggests a significant gap in the current understanding of factors influencing teacher well-being and performance.

Incorporating the role of job satisfaction as a mediator in the relationship between work engagement and job performance is essential for a comprehensive understanding of the dynamics at play (Zang and Feng, 2023). Job satisfaction, conceptualized as an emotional assessment of one’s job, serves as a crucial intermediary that shapes how engagement translates into performance outcomes. Despite the acknowledged impact of work engagement on various facets of job-related success, the literature has often overlooked the nuanced mechanism through which this process occurs. Specifically, there is a paucity of research investigating how the emotional evaluation of one’s job satisfaction mediates the path from engagement to enhanced job performance. This gap is significant because job satisfaction encompasses a broad range of emotional responses to one’s work environment, duties, and expectations, which in turn can influence motivation, productivity, and ultimately, the quality of educational outcomes (Kumar and Indora, 2023).

Acknowledging job satisfaction as a mediating factor allows for a deeper exploration of the psychological processes that underlie teacher behavior and effectiveness. It posits that the positive emotions and fulfillment derived from job satisfaction can amplify the positive effects of engagement on performance. Conversely, the absence of job satisfaction might mitigate these benefits, highlighting the complex interplay between these variables. Thus, this study seeks to empirically investigate the mediating role of job satisfaction in the engagement-performance nexus within the context of Chinese preschool education. By doing so, it aims to unravel the emotional and psychological mechanisms that facilitate the translation of teacher engagement into tangible improvements in job performance. This nuanced understanding could offer pivotal insights for developing targeted interventions aimed at boosting both teacher well-being and effectiveness, thereby contributing significantly to the body of knowledge on teacher engagement and performance.

This study aims to bridge these gaps by investigating the nuanced pathways through which work engagement affects job performance in Chinese preschool teachers, with a specific focus on the roles of emotional experiences and job satisfaction. By doing so, it seeks to offer empirical evidence that can inform the development of targeted interventions and support mechanisms designed to enhance the educational work environment. Moreover, the research aims to have broader implications for educational policy and administration, providing insights that can guide the formulation of more effective professional development programs and resource allocation strategies. The interconnection between teacher satisfaction and student achievement underscores the critical nature of this investigation.

Additionally, by concentrating on the Chinese educational context, this study endeavors to contribute to the global discourse on teaching dynamics. It aims to provide valuable insights for cross-cultural comparisons, enhancing the understanding of educational practices and policies from a perspective that integrates universal principles with culturally specific elements. Through this approach, the research seeks to address the significant lacunae in the literature, offering a comprehensive analysis of the factors influencing teacher engagement and performance in preschool settings.

## Literature review and hypotheses development

### Chinese preschool teachers’ engagement and its impact on personal and students’ outcomes

Work engagement plays a relevant role in educational research, and received increasingly attention in the Preschool Teachers’ field (Mazzetti et al., 2023). The relevance of preschool teaching in shaping the early learning and social development of children cannot be overstated (Zang and Feng, 2023). Early childhood is a critical period for cognitive, emotional, and social development. Preschool teachers, therefore, play a central role in influencing these developmental trajectories. Engaged teachers, who exhibit high levels of energy and involvement in their work, are more likely to create stimulating and nurturing learning environments (Wartenberg et al., 2023). This, in turn, fosters a positive developmental way conducive to the holistic growth of young learners. In the Chinese context, where education is highly valued, the role of the preschool teacher becomes even more significant.

The JD-R model, initially conceptualized by Bakker and Demerouti (2007, 2017), provides a theoretical foundation for understanding work engagement. At its core, the model posits that job demands (Bakker et al., 2023; Demerouti and Bakker, 2023), when balanced with adequate resources, can foster high levels of work engagement (Juliana et al., 2021). This heightened level of engagement, characterized by vigor, dedication, and absorption in work, is crucial in navigating the demanding environment of preschool education.

In the context of Chinese preschool education, work engagement embodies a motivational state that not only has a consequential impact on students’ outcomes, but also on personal wellbeing and institutional objectives. In China, where the educational landscape is rapidly evolving, the well-being of preschool teachers has garnered increased attention (Gao et al., 2023). This shift in focus is not merely about enhancing job satisfaction but also about recognizing teachers as workers whose personal well-being is linked to their professional efficacy and, by extension, to the developmental outcomes of their students.

### Affective neuroscience and primary emotions

While substantial empirical research has delved into the impacts of work engagement across personal, student, and institutional outcomes, the specific mechanisms underlying these effects remain partially understood (Mazzetti and Schaufeli, 2022). Recent advancements highlight the critical mediating role of emotions in the dynamics between job characteristics and work-related outcomes (Hetland et al., 2023). Evidenced by various studies, both positive and negative emotions have been shown to mediate relationships between perceived justice and counterproductive work behaviors, stress levels (Chu, 2014), the effectiveness of Human Resource Management practices on contextual performance (Edgar et al., 2018), and the influence of job insecurity on extra-role behaviors (Khan et al., 2013), among other factors (Le Roy et al., 2012).

The conceptualization of basic emotions, fundamental to understanding human behavior and personality, has been significantly advanced by the seminal works of Paul Ekman and Robert Plutchik. Ekman’s research identified six basic emotions—sadness, happiness, fear, anger, surprise, and disgust—that are universally recognized across cultures (Ekman, 1992, 1999). These emotions, according to Ekman, are inherent, serving adaptive evolutionary functions and leading to distinct behavioral expressions (Ekman, 1992). Similarly, Plutchik proposed a model of eight primary emotions: anger, fear, sadness, disgust, surprise, anticipation, trust, and joy. His framework, known as the wheel of emotions, illustrates how basic emotions combine to form complex feelings, emphasizing the adaptive and evolutionary roles of these emotional responses (Plutchik, 2001).

Both Ekman and Plutchik underscore the universality and categorical nature of basic emotions. They argue that these emotions solve universal adaptive problems and are discrete, leading to specific behaviors. This understanding is critical, suggesting that despite differences in emotional expression influenced by cultural or social factors, the core functions and categories of basic emotions remain consistent across human societies.

Building on this foundational understanding of basic emotions, Affective Neuroscience theory, as proposed by Panksepp (2013), offers an explanatory model centered around primary emotional systems embedded within specific neural circuits. Unlike descriptive models that categorize emotions based on behavioral expressions or cultural constructs, Affective Neuroscience seeks to understand the biological underpinnings of these emotional states. Panksepp identified several primary emotional systems—PLAYFULNESS, CARING, SEEKING, FEAR, ANGER, and SADNESS—that are foundational to psychological well-being and behavior (Panksepp, 1982, 2004, 2005). These systems are linked to specific neural networks and provide insights into the biological basis of personality traits and their influence on behavior.

The Affective Neuroscience Personality Scales (ANPS) operationalize Panksepp’s theory by measuring individual differences in these primary emotional systems. The ANPS assesses affective dispositions such as CARING, indicative of nurturing tendencies, and SEEKING, reflecting a drive towards exploration and positive engagement with the environment, alongside dimensions representing negative affect such as ANGER and FEAR (Davis et al., 2003). A current debate persists between affective and cognitive neuroscientists regarding these concepts (Panksepp et al., 2017). However, the ANPS has been translated into multiple languages and undergone cross-cultural validation, showing associations with the Big Five Model of personality (Özkarar-Gradwohl, 2019). The Positive Affect component of the ANPS includes emotions like CARING, defined as the propensity to care for others, and SEEKING, characterized by a positive outlook towards new experiences and a sense of resilience. Conversely, the Negative Affect component encompasses emotions like ANGER, marked by feelings of frustration and expressive anger, and FEAR, which involves feelings of anxiety and worry (Panksepp, 2005).

This study, grounded in the Affective Neuroscience theory, posits the mediating role of primary emotions, specifically CARING, SEEKING, ANGER, and FEAR, in the relationship between work engagement and its outcomes. The hypothesis suggests that both positive and negative emotions, as conceptualized by Affective Neuroscience, act as individual differences in emotionality and have a mediating influence on job-related outcomes.

This study employs the Affective Neuroscience framework to explore how primary emotions mediate the relationship between work engagement and its outcomes. By integrating the universal and adaptive perspectives on basic emotions provided by Ekman and Plutchik with Panksepp’s biological and neural-based approach, this research hypothesizes that a spectrum of positive to negative emotions influences individual differences in emotionality, significantly impacting job-related outcomes (Tracy and Randles, 2011).

The choice to prioritize Jaak Panksepp’s Affective Neuroscience classification over Paul Ekman’s categorization of basic emotions in our study is driven by the theoretical and methodological alignment of Panksepp’s framework with the objectives and approaches of our research. While both classifications offer valuable insights into the nature of emotions, Panksepp’s model provides a more direct connection to the underlying biological and neurological mechanisms that influence behavior and personality, which is central to our study’s focus.

Firstly, Panksepp’s Affective Neuroscience theory offers a deep biological and neuroscientific foundation for understanding emotions. This theory delineates primary emotional systems—such as SEEKING, FEAR, ANGER, and SADNESS—not merely as psychological constructs but as neural processes rooted in brain function. Panksepp’s approach provides insights into how these emotional systems influence behavior from a neurobiological perspective, making it particularly relevant for exploring the intricate relationships between personality traits and job performance in educators. This biological grounding is essential for the study’s aim to link affective traits with tangible outcomes in an educational setting, offering a mechanistic understanding of how emotions drive behavior.

On the other hand, Ekman’s classification focuses on the universality of facial expressions associated with six basic emotions—happiness, sadness, fear, anger, surprise, and disgust. While invaluable for studies on emotional expression and recognition across cultures, Ekman’s model is less equipped to delve into the neurobiological underpinnings of emotional experiences and their impact on job performance and well-being. The emphasis on facial expressions and the external manifestation of emotions, central to Ekman’s approach, does not align as closely with the objectives of a study aimed at understanding the internal emotional processes that mediate work engagement and outcomes.

Furthermore, the Affective Neuroscience perspective is particularly suited to exploring the mediating role of emotions in work engagement due to its focus on primary emotional systems as drivers of behavior. Panksepp’s classification allows for a nuanced exploration of how specific emotional tendencies, assessed through the Affective Neuroscience Personality Scales (ANPS), influence professional outcomes. This is crucial for a study focused on preschool teachers, where emotional dispositions can significantly impact teaching styles, classroom environments, and, ultimately, student well-being and learning outcomes.

In summary, while Ekman’s classification of basic emotions has significantly contributed to the field of emotion research, the Affective Neuroscience framework proposed by Panksepp is more appropriate for this study due to its emphasis on the neurobiological basis of emotions. This focus better facilitates the examination of the complex interplay between affective traits and the multifaceted aspects of teachers’ professional performance and well-being, offering a richer, more detailed understanding of these relationships.

### Emotions, moods, and affective tendencies or behaviors

The distinctions between emotions, moods, and affective tendencies or behaviors is essential for clarifying the scope and methodology of the study, particularly in light of the focus on measuring affective tendencies rather than emotions *per se*. This distinction plays a critical role in understanding the nature of the constructs being measured and their relevance to preschool teachers’ performance and well-being.

Emotions are typically intense, short-lived psychological and physiological responses to specific stimuli or events. They are immediate reactions that can be clearly linked to what triggered them, such as fear in response to a perceived threat. Emotions are characterized by their specificity and short duration, often leading to specific behaviors or actions in response to the immediate context (Plutchik, 2001).

Moods, in contrast, are more diffuse and enduring affective states that do not necessarily have a specific identifiable cause. Unlike emotions, moods can last for hours, days, or even longer, and they influence an individual’s overall perception and interpretation of their environment rather than eliciting a direct behavioral response to a particular event. Moods can subtly color a wide range of experiences and interactions throughout their duration (Sander and Nummenmaa, 2021).

Affective tendencies, or personality traits related to emotionality, refer to stable predispositions towards experiencing certain moods or emotions more frequently or intensely than others. These tendencies shape an individual’s overall affective landscape and influence how they typically respond to a variety of situations over time. Affective tendencies are measured as enduring characteristics of an individual, rather than transient states like emotions or the more prolonged states of moods (Panksepp et al., 2017).

In the context of this study, the emphasis on measuring affective tendencies rather than discrete emotional states is deliberate and methodologically appropriate. Given the study’s focus on preschool teachers’ performance and well-being, it is the enduring affective traits that are of particular interest. These traits, such as the propensity for nurturing (CARING) or the drive for exploration and positive engagement (SEEKING), influence how teachers interact with their students and colleagues, shape the classroom environment, and ultimately impact their professional effectiveness and satisfaction.

Understanding that the study measures affective tendencies provides clarity on its aim to explore the foundational emotional dispositions that underpin behavior over time, rather than the immediate reactions to specific events or the fluctuating mood states that may also affect performance and well-being. This approach aligns with the theoretical framework of Affective Neuroscience, which posits that primary emotional systems are key drivers of behavior and well-being, offering a nuanced perspective on how affective traits impact professional outcomes in the educational sector.

### Job satisfaction and its mediating role into the relationships between engagement and performance

Job satisfaction, a subject of extensive research, is defined as a positive overall assessment of one’s job (Williams and Anderson, 1991). This assessment arises from concrete job features like financial rewards, task characteristics, and social relationships, as well as an emotional response to these aspects. Researchers have identified two distinct components of job satisfaction: an affective component and a cognitive component (Judge et al., 2017). The affective component is seen as a result of a rational comparison between actual working conditions and one’s expectations or desires. The cognitive component, on the other hand, represents a more general, overall positive emotional response to one’s job.

The relationship between the affective and cognitive aspects of job satisfaction has been a topic of debate. Thompson and Phua (2012) discussed the conceptual and statistical links between these components, indicating some controversy in the field. Nevertheless, there is a general agreement on the presence of both affective and cognitive factors in job satisfaction.

Job satisfaction might play a mediating role between positive workplace attitudes, such as work engagement, and job performance (Pranoto and Nuzulia, 2023; Wartenberg et al., 2023). This mediation is initially driven by emotions. Engaged workers are likely more motivated to alleviate stressful demands and enhance job resources (Yu et al., 2021). This process leads to more positive emotions and a reduction in negative affect, which in turn influences job satisfaction and subsequently impacts job performance (Zang and Feng, 2023).

The primary aim of the study is to examine a sequential mediating model in the context of Chinese preschool teachers, exploring the relationships between work engagement, emotions, job satisfaction, and job performance. This model initiates with work engagement as the starting point, hypothesizing that the level of engagement among Chinese preschool teachers significantly influences their emotional experiences, encompassing both positive and negative emotions. Following this, the study seeks to understand how these emotional states, as a result of work engagement, subsequently affect the teachers’ job satisfaction. The final link in this model is the impact of this job satisfaction on job performance.

Based on the revised literature, the following hypotheses are proposed:

*Hypothesis 1*: Work engagement will have a positive and significant influence on Job performance.

*Hypothesis 2*: The Positive Affect (CARING, and SEEKING), and the Negative Affect (ANGER, and FEAR) mediated the relationship between Work engagement and Job performance.

**Figure 1:**
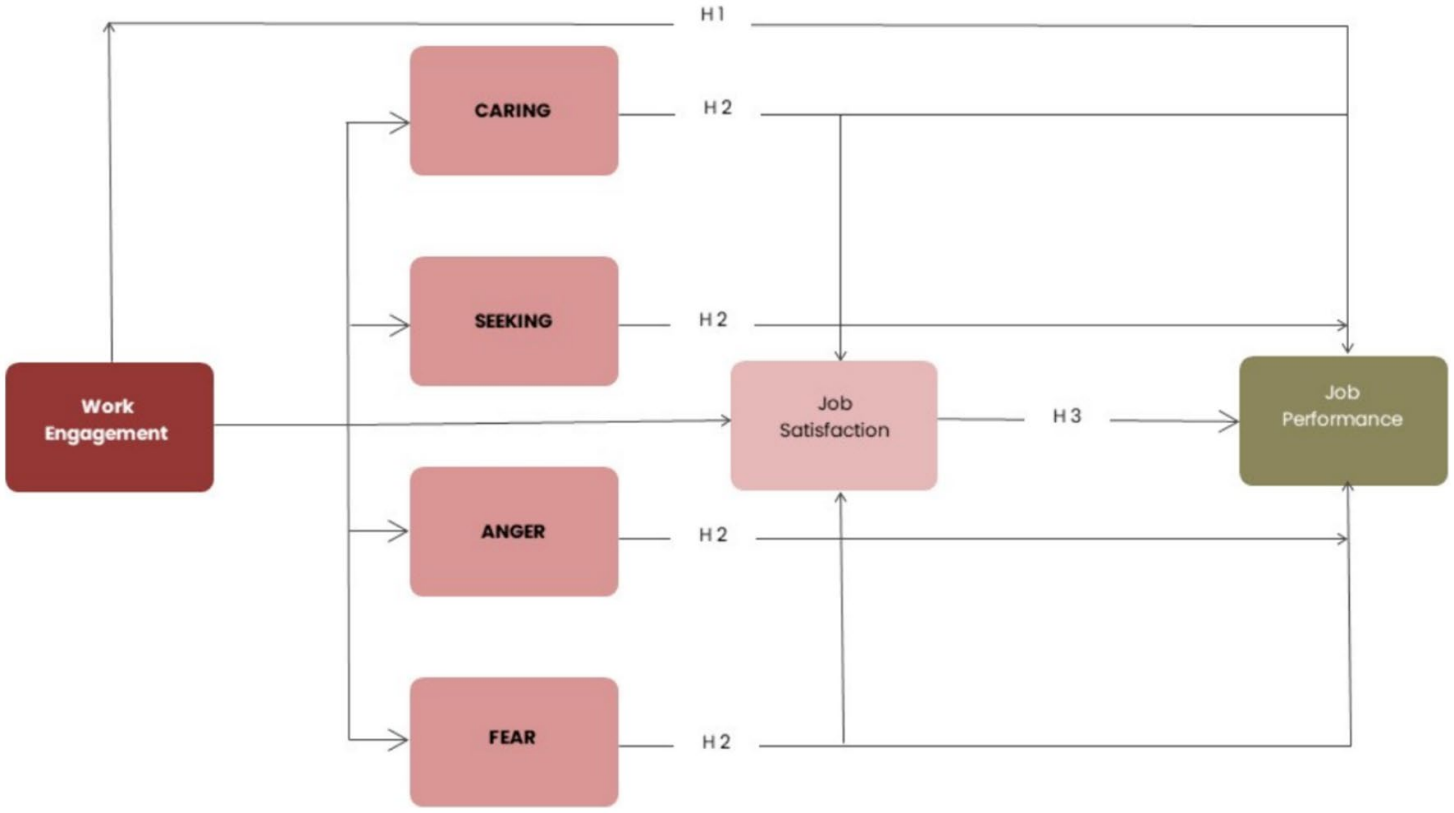
Research model with hypotheses.

*Hypotheses 3*: Job satisfaction mediates the relationship between Work Engagement and Job Performance, previously mediated by Positive and Negative affect. The full research model is depicted in Figure 1.

To sum up, the primary contribution would be providing information that could enhance teacher work engagement, emotional well-being, job satisfaction, and performance, with broader implications for educational practices, policy development, and cross-cultural understanding in the field of early childhood education.

## Method

### Participants and procedure

This study’s sample comprised 842 Chinese preschool teachers, drawn from a variety of settings including public and private institutions, kindergartens, and community or home-based preschools in several local communities. The gender distribution within the sample was notably imbalanced, with females constituting 74.1%. This reflects the broader trend in China’s preschool teaching workforce, which is predominantly female. The average age of the participants was 39.65 years, with a standard deviation (SD) of 11.98. In terms of professional experience, the average number of teaching years was 12.81 (SD = 12.96), and the average duration of employment at their current school was 7.841 years (SD = 11.43).

Participants were recruited for a time-lagged study examining Personal Wellbeing and Performance. Recruitment channels included popular social media platforms such as WeChat and Weibo, online forums, social media groups, and professional networks like LinkedIn. The recruitment message outlined the study’s objectives and provided a link to the initial survey. Participants do not receive any financial or material incentives for their answers. The time-lagged design was employed to facilitate shorter surveys at each point, optimized for mobile devices, with concise, easy-to-navigate questions.

The survey was administered through Wenjuanxing, an online platform, across four time points: Time 1 (T1, September 2022), Time 2 (T2, February 2023), Time 3 (T3, March 2023), and Time 4 (T4, July 2023). Prior to commencement, the study received ethical approval from the Ethical Committee of Southwest University. The initial section of the T1 survey provided information about anonymity, the voluntary nature of participation, the right to withdraw at any time, and the procedures for data analysis. Participants expressed their informed consent by responding affirmatively to a specific question. They were also asked to provide contact information for follow-up surveys. Participant numbers varied across the four time points, with initial participation at T1 being 1,211 and decreasing to 936 by T4, indicating participant attrition. Regarding the education, 36% of the sample have completed a Bachelor Degree, and 21.3% have a Master Degree and 5.9% have a Ph.D., while 1.7% were missing data.

### Instruments

#### Teachers’ work engagement (T1)

This variable was assessed by the Utrecht Work Engagement Scale (UWES-3) (Schaufeli et al., 2017), a shorter version of the original scale, consisting of three items. Each of these items represents one dimension of work engagement: vigor (*At my work, I feel bursting with energy*), dedication (*I am enthusiastic about my job*), and absorption (*I am immersed in my work*). Respondents rate these statements on a Likert scale ranging from 1 (*Never*) to 5 (*Always*). Despite its brevity, the UWES-3 has demonstrated good psychometric properties, including validity and reliability, in various studies, as well as it has been used to provide a global assessment of Work Engagement.

#### Positive and negative emotions (T2)

These variables have been assessed using four of the six dimensions of the Affective Neuroscience Personality Scales (ANPS), by the Chinese version (Sindermann et al., 2018). CARING (6 items) and SEEKING (6 items) were used for Positive affect, while ANGER (6 items), and FEAR (6 items) for Negative affect. The range of answer options was 1 (*Strongly disagree*) to 5 (*Strongly agree*). The full scale in Chinese is included as Table 1.

**Table 1:** Full scale of items of the affective neuroscience personality scales (ANPS), and utrecht work engagement scale (UWES-3), English and Chinese versions.

Affective neuroscience personality scales (ANPS)	
SEEKING	寻找
I really enjoy looking forward to new experiences.	我非常喜欢期待新的体验。
I am usually not highly curious.	我通常好奇心不强。
My curiosity often drives me to do things.	我的好奇心常常促使我去做事。
I rarely feel the need just to get out and explore things.	我很少觉得有必要走出去探索事物。
Whenever I am in a new place, I like to explore the area and get a better feel for my surroundings.	每当我到了一个新地方，我都喜欢去探索一下，以便更好地了解周围的环境。
I am not an extremely inquisitive person.	我不是一个好奇心极强的人。
CARING	关爱
I often feel a strong need to take care of others.	我经常强烈地需要照顾他人。
I like taking care of children.	我喜欢照顾孩子。
Caring for a sick person would be a burden for me.	照顾病人对我来说是一种负担。
I do not especially like being around children.	我不特别喜欢和孩子在一起。
I am a person who strongly feels the pain of other people.	我是一个能强烈感受到他人痛苦的人。
I am not particularly affectionate.	我没有特别的感情。
ANGER	愤怒
When I am frustrated, I usually get angry.	当我感到沮丧时，我通常会生气。
My friends would probably describe me as hotheaded.	我的朋友可能会说我是急性子。
When I am frustrated, I rarely become angry.	当我感到沮丧时，我很少生气。
People who know me well would say I almost never become angry.	熟悉我的人会说我几乎从不生气。
I hardly ever become so angry at someone that I feel like yelling at them.	我几乎从来没有对一个人生气到想对他大喊大叫的地步。
When people irritate me, I rarely feel the urge to say nasty things to them.	当别人激怒我时，我很少有冲动对他们说难听的话。
FEAR	恐惧
People who know me well would say I am an anxious person.	熟悉我的人会说我是一个焦虑的人
I am not frequently jittery and nervous.	我不会经常紧张不安。
I would not describe myself as a worrier.	我不会把自己描述成一个爱操心的人。
I have very few fears in my life.	我的生活中很少有恐惧。
My friends would say that it takes a lot to frighten me.	我的朋友会说，要让我害怕，需要付出很多代价。
There are very few things that make me anxious.	让我焦虑的事情很少。

Utrecht work engagement scale (UWES-3)	
At my work, I feel bursting with energy	在工作中，我感到精力充沛
I am enthusiastic about my job	我对工作充满热情
I am immersed in my work	我沉浸在工作中

#### Job satisfaction (T3)

This variable was assessed using the Job satisfaction scale (Shi et al., 2014), including 11 items, that cover both the intrinsic and the extrinsic facets of Job satisfaction. Intrinsic factors are those related to tasks and responsibilities associated with a concrete job, while the extrinsic are hose related to the working environment. The scale was rated using a five-point ordinal scale ranging from 1 (*Dissatisfied*) to 5 (*Very satisfied*).

#### Job performance (T4)

We measured job performance using the adapted Chinese version of the Williams and Anderson’s (1991) measure. It includes five items, rated on a scale from 1 (*Strongly disagree*) to 5 (*Strongly agree*). This measure taps job-focused contributions (*Adequately completes assigned duties*; *I can competently complete assigned work*, *I can perform the duties of my job description*, and *I never neglect my job responsibilities*).

### Data analyses

The statistical analyses of our study were performed using the Statistical Package for the Social Sciences (SPSS). To examine the relationships between work engagement, job satisfaction, and job performance, as well as the mediating role of job satisfaction in the engagement-performance link, we employed the PROCESS macro for SPSS, as developed by Hayes (2022). Specifically, we utilized Model 80 of the PROCESS macro, which is designed for complex mediational analyses, including the examination of direct and indirect effects within a proposed model.

In conducting our analyses, we first ensured that the data met the necessary assumptions for mediation analysis. The PROCESS macro employs bootstrapping, a non-parametric resampling procedure, to test the indirect effects. For our study, we used 5,000 bootstrap samples to generate bias-corrected confidence intervals for the indirect effects of work engagement on job performance through job satisfaction. This bootstrapping method provides a robust estimate of indirect effects and their associated confidence intervals without relying on the assumption of normality of the sampling distribution.

The mediation hypotheses are supported if the zero is not included in the 95% bias-corrected confidence interval for the indirect effect, indicating that the effect is statistically significant at *p* < 0.05. This approach to mediation analysis allows for a comprehensive examination of how job satisfaction mediates the relationship between work engagement and job performance, providing insights into the underlying mechanisms of this relationship.

By employing the PROCESS macro Model 80 and utilizing 5,000 bootstrap resamples for our analysis, we aimed to ensure the robustness and reliability of our findings. This detailed methodology provides a clear understanding of the analytical procedures employed and the basis for the conclusions drawn from our study.

## Results

### Correlational analyses

As presented in Table 1, teachers reported high levels of Work Engagement, Job satisfaction and SEEKING, but medium levels of CARING and Job Performance, as well as lower levels of Negative affect (both ANGER and FEAR). Concurrently, there was a significant positive correlation between Work Engagement and Job Satisfaction, and a negative one with FEAR. As anticipated, Job Satisfaction demonstrated a significant positive association with Positive affect, and negative association with ANGER and FEAR.

### Mediating chain model

*Hypothesis 1*: Work engagement influence on Job performance.

We performed a chain mediating effect test using Hayes’ Process model 80. The results showed that *R* = 0.6017, R-sq = 0.3621, *F* = 78.98, df1 = 6.00, df2 = 835.00, MSE = 0.3309, *p* = 0.0000, indicating that the baseline model was a good fit and the model and the results could be adopted. The total effect of X on Y was 0.3137, *p* < 0.001. Further analysis of the results showed that the direct effect of Work Engagement on Job Performance was 0.1810, SE = 0.0459, *t* = 3.9427, *p* < 0.001, and the effect result do not contain 0 (LLCI = −0.0909, ULCI = 0.2711), supporting H1.

*Hypothesis 2*: Positive Affect (CARING, and SEEKING), and Negative Affect (ANGER, and FEAR) mediated between Work engagement and Job performance.

Examining the other sets of results on mediators, indicated that the model was a fully mediated effects model. The direct effect of Work Engagement on CARING was also statistically significant [*b* = −0.2371, SE = 0.0448, *p* < 0.001, 95% CI (0.14, 0.32)], as well as on SEEKING [*b* = 0.3844, SE = 0.0285, *p* < 0.001, 95% CI (0.32, 0.44)], and FEAR [*b* = −0.1272, SE = 0.0411, *p* < 0.05, 95% CI (−0.20, −0.04)]. On the contrary, the direct effect of Work Engagement on ANGER [*b* = −0.0771, SE = 0.0520, *p* < 0.001, 95% CI (−0.17, 0.02)] was not significant. This implies that the effect of Work Engagement on Job performance is mediated by the other variables in the model, except of ANGER, partially supporting H2.

*Hypotheses 3*: Job satisfaction mediates between Work Engagement and Job Performance, previously mediated by Positive and Negative affect.

Further analysis of each chain of influence revealed the following findings. Job Satisfaction mediated the effect of Work Engagement on the Job performance of teachers with an effect size of 0.0726 (LLCI = −0.0203, ULCI = 0.1655), and therefore H3 was not fully supported. CARING increased Job Performance, with an effect size of 0.4165, and this effect was fully significant, providing additional support to H2. CARING increased Job satisfaction and ultimately influenced Job performance, with an effect size of 0.0940 (*p* < 0.001), supporting H3. SEEKING increased Job Performance, with an effect size of 0.0554, but this effect was not significant, failing to support H2. SEEKING increased Job satisfaction and ultimately influenced Job performance, with an effect size of 0.0611, and this effect was significant (*p* < 0.05), providing additional support to H3. ANGER increased Job Performance, with an effect size of 0.0554, but this effect was not significant, failing to support H2. ANGER decreased Job satisfaction and ultimately influenced Job performance, with an effect size of −0.1151, and this effect was fully significant, providing additional support to H3. Finally, FEAR increased Job Performance, with an effect size of 0.0371, but this effect was not significant, failing to support H2. FEAR decreased Job satisfaction and ultimately influenced Job performance, with an effect size of −0.0284, but this effect was not significant, failing to support H3. Details in Table 2.

**Table 2:** Descriptive statistics and Pearson’s matrix correlation.

	Mean	S.D.	WE	C	S	A	F	JS
Work engagement (T1)	4.12	0.65	1					
Caring (T2)	3.22	0.86	0.179^**^	1				
Seeking (T2)	4.35	0.59	0.422^**^	0.288^**^	1			
Anger (T2)	2.82	0.99	−0.051	−0.086^*^	−0.022	1		
Fear (T2)	1.84	0.78	−0.106^**^	−0.240^**^	−0.234^**^	0.176^**^	1	
Job satisfaction (T3)	4.10	0.65	0.727^**^	0.284^**^	0.386^**^	−0.227^**^	−0.179^**^	1
Job performance (T4)	3.42	0.71	0.314^**^	0.546^**^	0.275^**^	0.050	−0.101^**^	0.315^**^

*N* = 842. WE, work engagement; C, Caring; S, SEEKING; A, ANGER; F, FEAR; JS, job satisfaction. ^*^p < 0.05; ^**^p < 0.01.

**Figure 2:**
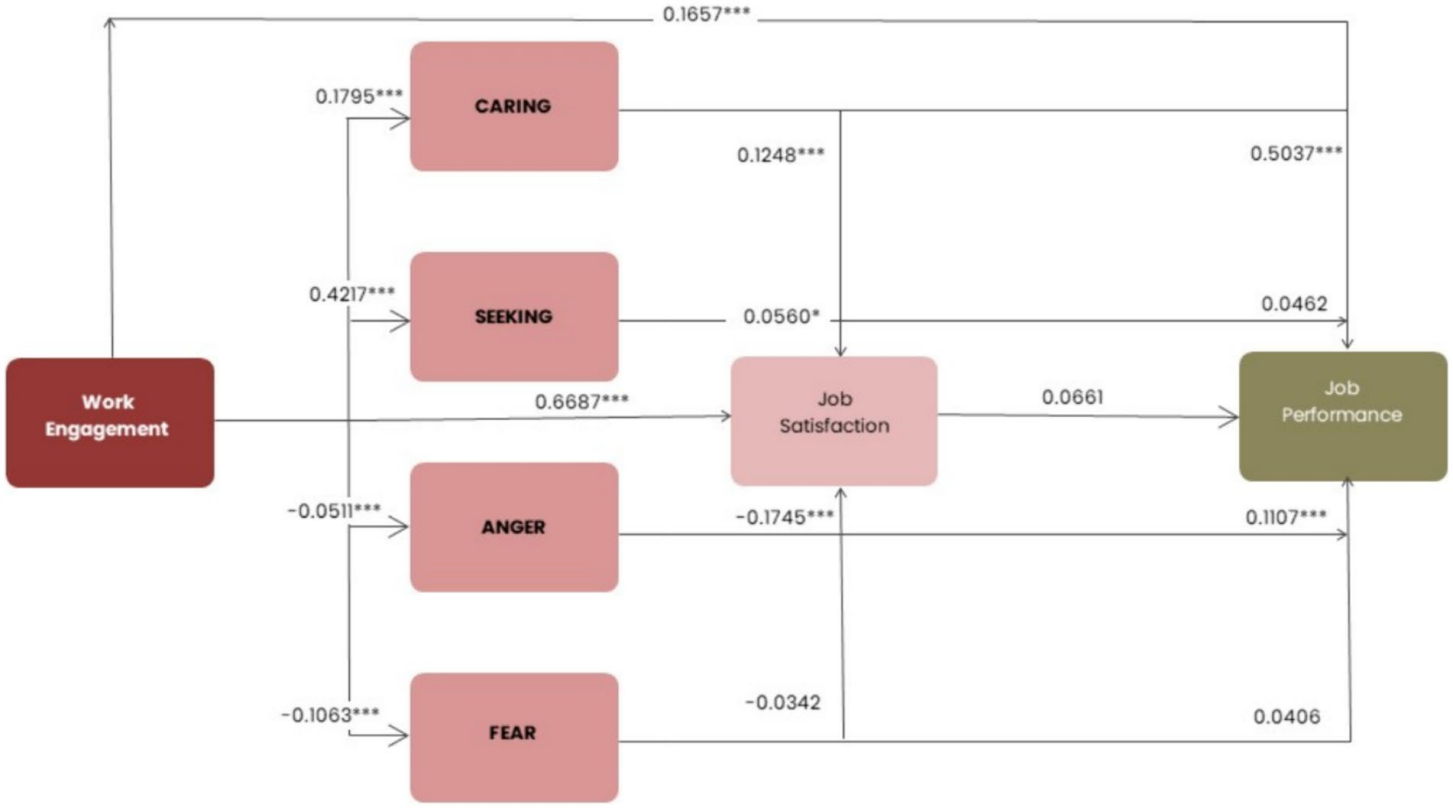
Standardized effects sizes for the mediating chain model.

It is important to note that SEEKING and FEAR did not mediate Work Engagement and Job Performance alone, but SEEKING enhanced Job Performance by increasing Job satisfaction. In contrast, CARING and ANGER not only mediated Work Engagement and Job performance directly, but also enhanced Job Performance by increasing Job satisfaction. But, in terms of effect size, CARING had the unique significantly indirect effect, which reflects positive affective influences that impact on Job satisfaction, later improving Job Performance. Details in Figure 2.

## Discussion

The present study has a main aim to test the mediating chain model where Job satisfaction mediates the relationships between Work engagement, previously mediated by emotions (CARING, SEEKING, ANGER and FEAR), and Job performance. Our findings also seek to provide preliminary support for the empirical evidence on the Affective Neuroscience research into the educational field (Davis and Montag, 2019). Firstly, the study’s correlational matrix suggested significant covariation between emotional states and professional outcomes in the teaching context. High levels of work engagement, job satisfaction, and SEEKING behavior among teachers are positively correlated. This implies that engaged teachers, who are proactive in their professional approach, tend to experience higher job satisfaction. These findings agreed with a wide range of studies testing the relationships between Work engagement and Job Satisfaction (Mazzetti et al., 2023). Among other, a recent study among hospitality instructors in India, that supported the correlation between job satisfaction and work engagement (Kumar and Indora, 2023). This suggests a consistent trend across different educational settings where engagement significantly correlates job satisfaction. In a similar vein, a research also conducted with Chinese preschool teachers suggested that job satisfaction positively related to Work engagement (Zang and Feng, 2023), despite that the cross-sectional design of that study precludes to assume causation.

Contrastingly, the negative correlation between work engagement and FEAR suggests that higher engagement in work is associated with lower levels of fear. This finding is significant as it highlights the potential of work engagement as a buffer against negative emotions in a stressful profession like teaching (Mazzetti and Schaufeli, 2022). The moderate levels of CARING and job performance, coupled with lower levels of negative affect, particularly ANGER and FEAR, suggest a complex emotional landscape in teaching. While teachers exhibit a certain level of empathy and concern for others, these traits do not directly correlate with higher job performance. This indicates that while emotional competencies are important in teaching, they are not the sole concomitants of job performance. In line with the present study, the role of emotional states in job satisfaction and engagement has been explored by Butakor et al. (2021), who found that emotional intelligence positively affects job satisfaction and, consequently, professional identity in Ghanaian teachers. Those findings align with the current study on the impact of emotions like CARING and FEAR on job satisfaction and performance.

Secondly, the study’s mediating chain model showed that a significant direct effect of work engagement on job performance confirms the importance of engagement in predicting job performance. However, the lack of a significant direct effect of work engagement on ANGER suggests that engagement does not necessarily mitigate all forms of negative affect. Moreover, the mediating roles of CARING, SEEKING, ANGER, and FEAR in the relationship between work engagement and job performance are the most relevant contribution of the present study. CARING and ANGER not only mediate this relationship directly but also enhance job performance by increasing job satisfaction. This suggests that the impact of these emotional states on job performance would be mediated through job satisfaction, highlighting the complex pattern of relationships involving satisfaction at work, as recent studies stated (Wang and Shi, 2023). However, contrasting views emerge in the context of negative emotions. For instance, the study on early childhood teachers conducted in India suggests that technostress and techno-overload are more influential, though their capacity to generate negative emotions, on job satisfaction compared to work–family conflict (Pranoto and Nuzulia, 2023). This contrasts with the current study where negative emotions like ANGER and FEAR showed significant but varied impacts on job satisfaction and performance, accordingly to recent findings (Hu et al., 2024).

At the same time, the current study’s findings on the mediating roles of emotional and psychological factors are supported by research like that of Buonomo et al. (2021), who found that compassion satisfaction, akin to CARING in the current study, is strongly related to engagement at school. However, their finding that compassion satisfaction does not mediate the relationship between ethical leadership and work engagement provides a divergent perspective compared to the current study’s broader approach to emotional mediation. The debate about the directionality of the influences between work engagement and other job-related constructs, as satisfaction and job crafting among others, continues evolving (Topa and Aranda-Carmena, 2022) as well as the amount of empirical evidence rapidly increases (Laguía et al., 2024). The implication seems clearing indicating that engagement in one’s work and a proactive attitude are key drivers of job satisfaction in educational settings.

The study’s findings are very relevant in understanding the emotional dynamics of teaching. Moreover, the present results partially confirmed the relationship between job satisfaction and professional outcomes such as turnover intentions, absenteeism, and teacher-student interactions, as found in the research synthesis by Wartenberg et al. (2023), provides a broader context for the current study. It suggests that job satisfaction is a critical factor in various aspects of teachers’ professional lives, beyond just job performance (see Table 3).

**Table 3:** Indirect effects of the mediating chain model.

Indirect effect	Effect	BootSE	BootLLCI	BootULCI
TOTAL	0.1617	0.0341	0.0924	0.2289
Ind1 WE- > C- > JP	0.0987	0.0178	0.0654	0.1344
Ind2 WE- > S- > JP	0.0213	0.0138	−0.0056	0.0497
Ind3 WE- > A- > JP	−0.0062	0.0047	−0.0171	0.0019
Ind4 WE- > F- > JP	−0.0047	0.0036	−0.0121	0.0016
Ind5 WE- > JS- > JP	0.0483	0.0283	−0.0077	0.1023
Ind6 WE- > C- > JS- > JP	0.0016	0.0011	−0.0003	0.0038
Ind7 WE- > S- > JS- > JP	0.0017	0.0014	−0.0004	0.0052
Ind8 WE- > A- > JS- > JP	0.0006	0.0006	−0.0003	0.0021
Ind9 WE- > F- > JS- > JP	0.0003	0.0003	−0.0001	0.0009

WE, work engagement; C, CARING; S, SEEKING; A, ANGER; F, FEAR; JS, Job satisfaction, JP, job performance.

### Limitations and suggestion for future research

As others, the present study has several limitations that need to be acknowledge. One significant limitation is the gender distribution of the sample. With females constituting 74.1% of participants, the findings are less applicable to male teachers or more gender-balanced teaching environments. This gender imbalance, reflective of China’s preschool teaching workforce, narrows the study’s generalizability. Moreover, the wide range in participants’ ages and teaching experience introduces variability in the data that could be influenced by different career stages or life experiences, rather than the variables under study. Regarding the recruitment method, it also presents a limitation. Participants were recruited through social media platforms and professional networks, which may introduce selection bias. This method likely over-represents individuals who are more engaged in these networks, potentially excluding less connected educators.

Additionally, the time-lagged design of the study, while reducing survey fatigue, led to participant attrition. The decreasing number of participants from the first to the last survey point raises concerns about attrition bias, where later data may not accurately represent the initial sample. Lastly, the study’s ethical considerations, including the requirement for contact information for follow-up surveys, might have influenced who chose to participate. Teachers with privacy concerns or reluctance to commit to a long-term study might have been less likely to participate, potentially skewing the sample. The approach to classifying emotions in this research, alongside the chosen survey instruments, presents intricacies that merit careful consideration. For instance, Panksepp and Watt (2011) acknowledged the dual role of the SEEKING system, which can be activated in contexts eliciting both positive and negative emotions. This duality introduces a layer of complexity to the analysis and interpretation of our findings, as the SEEKING emotion embodies a broad spectrum of emotional experiences, ranging from curiosity and exploratory zeal to anxiety-driven searching behaviors. This complexity underscores the challenge of neatly categorizing emotions that inherently possess multifaceted characteristics and can manifest across a wide range of psychological states.

Furthermore, the Affective Neuroscience Personality Scales (ANPS), while instrumental in identifying latent personality profiles (Zhu et al., 2007; Orri et al., 2017; Deak et al., 2024), blend aspects of emotions with their consequent affective tendencies or behaviors. This conflation has sparked debate, as emotions are traditionally viewed as distinct, immediate responses to stimuli, contrasting with moods, which are more diffuse, enduring affective states. The inclusion of such mixed categories in the ANPS reflects the ongoing discourse in affective science regarding the boundaries between emotions, moods, and the resultant affective dispositions. Overall, while the study offers valuable insights into the wellbeing and performance of Chinese preschool teachers, these limitations highlight the need for cautious interpretation of the findings and consideration of broader applicability in future.

### Suggestions for interventions with teachers

Interventions based on the evidence provided by the present study could be focused at the individual level, on teachers’ wellbeing and career development, as well as at the institutional level, on teachers’ retention.

For improving teacher wellbeing at the individual level, it’s important to focus on practical and direct measures. Professional development programs tailored to include stress management and emotional intelligence can be very effective. These programs should aim to equip teachers with the skills needed to handle the stresses of their job and understand their emotional responses better, thereby enhancing their overall wellbeing.

Providing mental health support is also crucial. Schools should offer services like counseling and stress management workshops. Regular mental health check-ins can help teachers deal with the emotional challenges of their job, particularly in managing negative emotions that can impact job performance (Xu et al., 2023).

On the institutional level, to improve teacher retention, schools can implement several strategies. Recognition and reward systems that acknowledge and appreciate teachers’ hard work can boost their morale and job satisfaction (Ma et al., 2023). This could include awards or bonuses for exceptional performance.

Creating opportunities for career advancement is also key. Teachers should have clear paths to progress in their careers, with chances for promotion and additional responsibilities. Knowing there are opportunities to grow and take on new challenges can motivate teachers and encourage them to stay with the institution longer.

Incorporating professional career development opportunities is critical for teachers’ growth and satisfaction. Schools should consider establishing a structured career development framework that allows teachers to identify and pursue their career goals within the education sector (Zhang and Jiang, 2023). This can include offering advanced training programs, workshops, and courses that not only focus on improving teaching methods but also cover areas like leadership, educational technology, and curriculum development. By investing in their professional growth, teachers feel valued and are more likely to remain engaged and committed to their institutions. Additionally, creating opportunities for teachers to assume leadership roles or participate in decision-making processes can be highly beneficial (Xiao and Tian, 2023). Teachers could be involved in curriculum development teams, school improvement initiatives, or mentorship programs for newer educators. These roles not only provide them with a sense of ownership and contribution beyond their classrooms but also pave the way for career advancement. Schools can also establish partnerships with universities or educational organizations to provide teachers with access to continued education and certification programs. These partnerships can offer pathways for teachers to gain additional qualifications, which can be instrumental in their career progression.

Policymakers should prioritize comprehensive teacher training and development. This includes not only enhancing pedagogical skills but also providing resources for emotional intelligence and stress management. Such training ensures teachers are well-equipped to meet the dynamic needs of education. Alongside, creating supportive work conditions is essential (Jiao et al., 2022). This can be achieved by reducing class sizes, managing workloads, and offering adequate administrative support, which collectively help in reducing teacher burnout and improving job satisfaction (Jia et al., 2022).

Additionally, policies should focus on clear career progression opportunities for teachers. This involves establishing leadership training programs and offering pathways for specialization and advancement. Ensuring competitive compensation is also vital for attracting and retaining qualified educators. Finally, implementing data-driven decision-making processes, where educational policies are shaped based on research and assessments, can lead to more effective educational strategies and outcomes.

## Conclusion

In conclusion, this paper significantly enhances our understanding of the relationship between emotional states, work engagement, and job performance in education. It thoroughly explores how different emotional factors like CARING, SEEKING, ANGER, and FEAR interact with job satisfaction and performance among teachers (Brienza et al., 2023). The study highlights the vital role of emotional skills and engagement in determining job performance and satisfaction. This research not only adds to the existing literature on teacher wellbeing and effectiveness but also provides important insights for educational policymakers and administrators. It points out the importance of focused interventions at both the individual and institutional levels to improve teacher wellbeing, career development, and retention. The findings of this paper are crucial for informing future educational policies and practices, ultimately contributing to the advancement of the educational sector.

## Data availability statement

The raw data supporting the conclusions of this article will be made available by the authors, without undue reservation.

## Ethics statement

The studies involving humans were approved by Ethical Board Committee of the Southwest University. The studies were conducted in accordance with the local legislation and institutional requirements. The participants provided their written informed consent to participate in this study.

## Author contributions

LL: Writing – original draft, Writing – review & editing. LJ: Writing – original draft, Writing – review & editing.
